# SPECT vs. PET in cardiac innervation imaging: clash of the titans

**DOI:** 10.1007/s40336-018-0289-4

**Published:** 2018-07-03

**Authors:** Rudolf A. Werner, Xinyu Chen, Mitsuru Hirano, Steven P. Rowe, Constantin Lapa, Mehrbod S. Javadi, Takahiro Higuchi

**Affiliations:** 1 Division of Nuclear Medicine and Molecular Imaging, The Russell H. Morgan Department of Radiology and Radiological Science, Johns Hopkins University School of Medicine, Baltimore, MD USA; 2 Department of Nuclear Medicine, University of Wuerzburg, Oberduerrbacher Strasse 6, 97080 Würzburg, Germany; 3 Comprehensive Heart Failure Center, University of Wuerzburg, Würzburg, Germany; 4 Department of Biomedical Imaging, National Cardiovascular and Cerebral Center, Suita, Japan

**Keywords:** ^18^F-LMI1195, ^11^C-hydroxyephedrine, ^123^I-metaiodobenzylguanidine, Positron emission tomography, Single photon emission computed tomography, Sympathetic nerve

## Abstract

**Purpose:**

We aim to provide an overview of the conventional single photon emission computed tomography (SPECT) and emerging positron emission tomography (PET) catecholamine analogue tracers for assessing myocardial nerve integrity, in particular focusing on ^18^F-labeled tracers.

**Results:**

Increasingly, the cardiac sympathetic nervous system (SNS) is being studied by non-invasive molecular imaging approaches. Forming the backbone of myocardial SNS imaging, the norepinephrine (NE) transporter at the sympathetic nerve terminal plays a crucial role for visualizing denervated myocardium: in particular, the single-photon-emitting NE analogue ^123^I-meta-Iodobenzylguanidine (^123^I-mIBG) has demonstrated favorable results in the identification of patients at a high risk for cardiac death. However, cardiac neuronal PET agents offer several advantages including improved spatio-temporal resolution and intrinsic quantifiability. Compared to their ^11^C-labeled counterparts with a short half-life (20.4 min), novel ^18^F-labeled PET imaging agents to assess myocardial nerve integrity have the potential to revolutionize the field of SNS molecular imaging. The longer half-life of ^18^F (109.8 min) allows for more flexibility in the study design and delivery from central cyclotron facilities to smaller hospitals may lead to further cost reduction. A great deal of progress has been made by the first in-human studies of such ^18^F-labeled SNS imaging agents. Moreover, dedicated animal platforms open avenues for further insights into the handling of radiolabeled catecholamine analogues at the sympathetic nerve terminal.

**Conclusions:**

^18^F-labeled imaging agents demonstrate key properties for mapping cardiac sympathetic nerve integrity and might outperform current SPECT-based or ^11^C-labeled tracers in the long run.

## Introduction

**Fig. 1:**
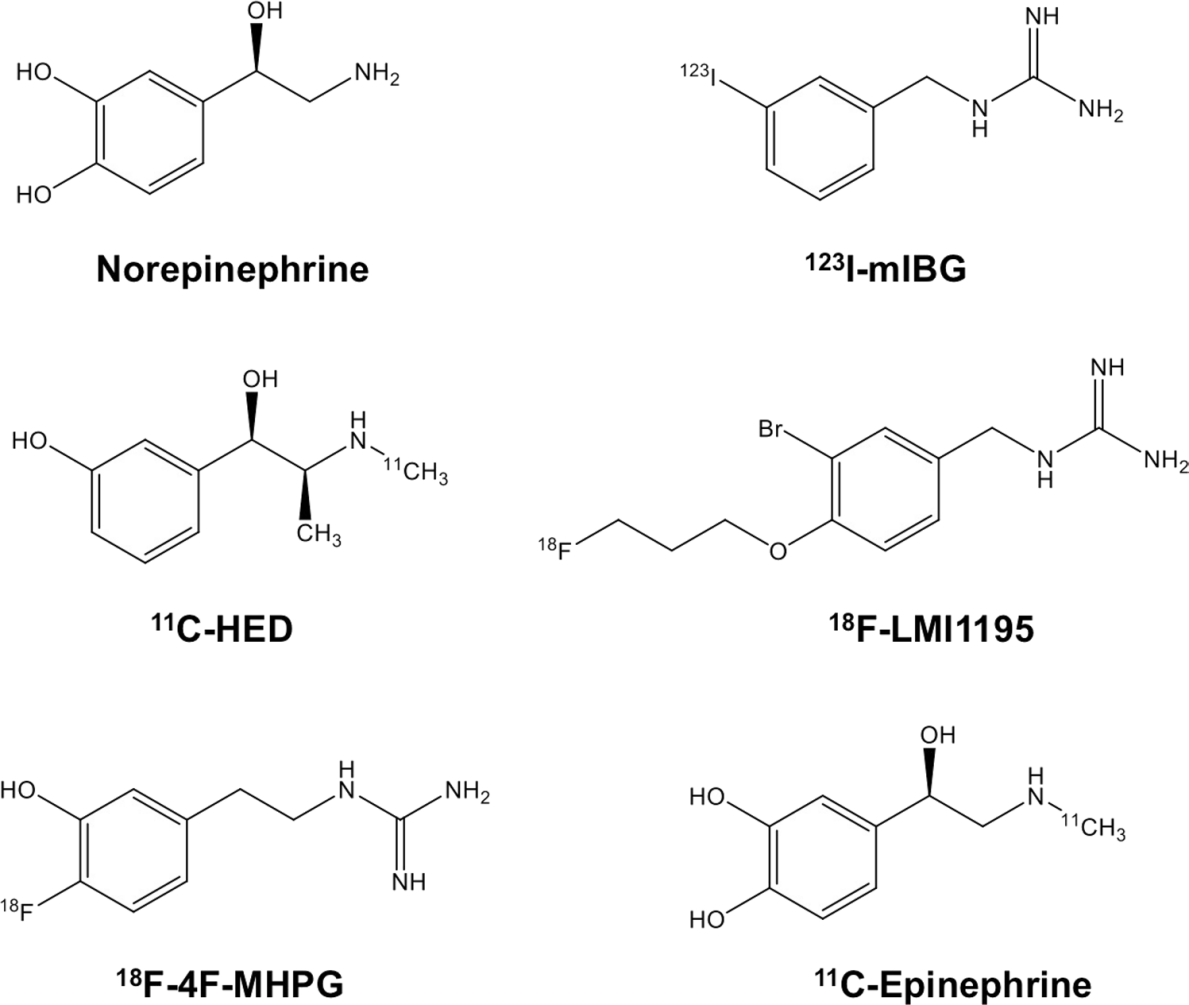
Chemical structures of radiolabeled catecholamine analogue tracers in comparison to physiological norepinephrine. ^123^I-meta-Iodobenzylguanidine (^123^I-mIBG), ^11^C-hydroxyephedrine (^11^C-HED), N-[3-Bromo-4-(3- [^18^F]fluoro-propoxy)-benzyl]-guanidine (^18^F-LMI1195), ^18^F-fluoro-3-hydroxyphenethylguanidine (^18^F-4F-MPHG)

Heart failure (HF) is primarily characterized by a vicious cycle: myocardial injury leads to reduced cardiac output, which triggers myocardial sympathetic nervous system (SNS) hyperactivity, which in turn leads to further cardiac damage and ultimately to a further reduction of systolic function [1, 2]. On a subcellular level, alterations of the myocardial SNS are manifest as an elevated plasma concentration of NE, an impaired function of NE transporter (uptake-1 mechanism) as well as by a reduced plasma clearance of NE in the synaptic cleft [3, 4]. This phenomenon of “cardiac NE spillover” causes severe damage to cardiac myocytes via cyclic AMP-mediated calcium overloads, which result in a decrease of synthetic activity and viability [5]. Apart from that, a continuous oversupply of NE at the sympathetic nerve terminal triggers remodeling of the left ventricle and the development of hypertensive left ventricular hypertrophy [6]. Not surprisingly, those insights into HF pathophysiology paved the way for a therapeutic approach via sympathetic inhibition: the CIBIS-II trial demonstrated a decreased cardiac mortality rate in HF patients undergoing treatment with adrenergic beta blockers [7]. Of note, an incremental cost effectiveness ratio compared to other conventional cardiovascular treatments was documented [8]. On the contrary, the investigators of the “Sustained-release moxonidine in patients with HF (MOXCON)” trial examined the beneficial role of generalized sympathetic inhibition in HF using the imidazoline receptor agonist moxonidine and an increase in death rate led to early termination of this promising approach in HF treatment [9]. Taken together, the delicate balance of risk and benefit of therapeutic interventions of cardiac sympathetic nerve blockade emphasizes the pivotal role of non-invasive imaging to assess the current status of the cardiac SNS. Moreover, such imaging concepts could potentially be applied to stratify one’s individual risk for sudden cardiac death or to determine the appropriate time-point at which to initiate further treatment for progressive HF. However, in contradistinction to conventional imaging modalities, molecular imaging using either single photon emission computed tomography (SPECT) or positron-emission tomography (PET) offers the unique opportunity to characterize alterations on a subcellular level and to gain deeper insights into cardiac disease development and early onset of HF [10]: Physiologic NE is stored in presynaptic vesicles and once a firing impulse has arrived at the nerve terminal, NE is exocitotically released into the synaptic cleft, where it is readily available for interacting with postsynaptic adrenoreceptors. After completing its primary task at the post-synapse, NE undergoes a recycling mechanism via the presynaptic NE transporter (uptake-1) and is stored in vesicles for potential re-use [10–12]. Comparable to its physiologic counterpart, radiolabeled NE analogues are taken up into the nerve terminals using the identical uptake-1 pathway and, therefore, impaired cardiac NE function is reflected by either decreased radiotracer uptake or increased tracer washout. Figure 1 summarizes commonly used catecholamine analogue tracers to assess cardiac sympathetic nerve integrity. In this review, we will discuss these radiolabeled SPECT and PET imaging agents for mapping the cardiac SNS, in particular focusing on recently introduced ^18^F-labeled PET imaging probes.

## Myocardial SNS imaging using SPECT

### ^123^I-meta-iodobenzylguanidine (^123^I-mIBG) for risk stratification of HF-related events

As a conventional scintigraphic approach, ^123^I-mIBG is a United States Food and Drug Administration-approved agent that has found widespread use in Japan, the United States, and Europe [13, 14]. Its prognostic role can be derived by calculating a heart-to-mediastinum ratio (HMR) obtained from planar scans, which can be considered as a semi-quantitative method to determine myocardial catecholamine uptake: patients below a certain HMR threshold are at a higher risk to experience cardiac events [15]. In a meta-analysis, Verberne et al. included 18 studies with a total of 1755 patients and concluded that decreased late HMR or increased myocardial ^123^I-mIBG washout are associated with a worse outcome compared to those patients without alterations in semi-quantitatively derived ^123^I-mIBG parameters [16]. These findings were further confirmed by the prospective “AdreView Myocardial Imaging for Risk Evaluation in Heart Failure (ADMIRE-HF)” trial, which emphasized the potential benefit of ^123^I-mIBG imaging for risk stratification in patients suffering from severe HF. A highly significant association between time of HF-related events and the HMR was derived, independent of other common clinical parameters, such as left ventricular ejection fraction (LVEF) or renal function [17]. In a similar vein, Nakajima et al. created dedicated mortality risk charts with ^123^I-mIBG imaging and clinical parameters (New York Heart Association Class (NYHA), Age, LVEF) with 2- and 5-year mortality risk estimations that aimed to provide a more flexible platform for short- and long-term therapeutic decision-making in patients suffering from congestive HF [18].

### ^123^I-mIBG as a risk stratification tool for potential device therapy candidates

Apart from risk stratification for HF-related cardiac events, the amount of denervated myocardium as assessed by ^123^I-mIBG is being increasingly utilized to guide the referring treating cardiologist in regards to what time to initiate further treatment, in particular by implantation of cardioverter defibrillators (ICD). ICD shocks for ventricular tachyarrhythmias are of utmost importance for secondary prevention of sudden cardiac death. Although the documented shock frequency varies among different studies [19], a considerable proportion of patients receive an inappropriate shock, in particular with single-chamber devices [20]. Apart from this risk of malfunction, up to 65% never have an appropriate ICD discharge after ICD implantation [21]. A recent published post hoc analysis performed by Verschure et al. demonstrated that the combination of late HMR and LVEF was associated with freedom of appropriate ICD therapy: consequently, it was concluded that ^123^I-mIBG scintigraphy might be helpful in excluding those patients who might not benefit from ICD implantation [22]. A combination of ^123^I-mIBG and the “Seattle Heart Failure Model” using demographic as well as clinical markers improved risk stratification in high-risk HF patients for potential therapeutic assessment with ICD or cardiac resynchronization therapy (CRT) [23]. The latter therapeutic approach has proven to be highly effective in patients with complete left bundle branch block, but an extensive body of literature demonstrated that a significant number of patients receiving CRT have to deal with device complications [24]. Thus, analogous to ICD therapy, ^123^I-mIBG imaging might meet this urgent clinical need of selecting appropriate candidates for CRT initiation. Utilizing a baseline ^123^I-mIBG scan, Nishioka et al. demonstrated that lower HMR could identify CRT non-responders [25]. The currently recruiting, event-driven Phase IIIb multicenter, randomized, prospective “International Study to Determine if AdreView Heart Function Scan Can be Used to Identify Patients With Mild or Moderate Heart Failure (HF) That Benefit From Implanted Medical Device (ADMIRE-ICD)” trial may provide further insights into the efficacy of ^123^I-mIBG for selecting patients who most likely benefit from ICD implantation [26]. Taken together, these data suggest that ^123^I-mIBG can be used to guide the treating cardiologist to identify high-risk patients who benefit from device therapy. Thus, one might speculate that the non-invasive assessment of myocardial nerve integrity using ^123^I-mIBG has the potential to outperform current selection criteria, such as LVEF or electrocardiogram-derived QRS duration [13, 27].

### ^123^I-mIBG for other clinical scenarios

Scintigraphic assessment of the SNS of the heart might be also helpful in heart-transplant recipients, as sympathetic reinnervation could successfully be monitored by ^123^I-mIBG up to 1 year after operation [28]. Thus, ^123^I-mIBG could have utility in selecting appropriate transplantation candidates or might contribute to further determining expected post-surgical survival rates [13]. Gerson et al. studied 343 diabetic and 618 nondiabetic subjects with NYHA class II or III HF and the combination of diabetes mellitus and HMR predicted HF progression independently. Thus, diabetic patients with impaired SNS function of the heart seem to be at higher risk and consequently, lifestyle changes could be further promoted or anti-diabetic treatment could be intensified among those individuals [29]. Apart from that, Sobajima et al. demonstrated immediate improvement of SNS activity (assessed by ^123^I-mIBG washout rate) after transcatheter aortic valve implantation (TAVI) in patients suffering from aortic valve stenosis. Hence, ^123^I-mIBG might serve as a risk stratification tool or could also monitor the therapeutic efficacy of TAVI [30]. Moreover, ^123^I-mIBG imaging has also been used to evaluate cardiac involvement in multiorgan diseases: On a small cohort of 5 patients, Yamamoto et al. demonstrated that cardiac sympathetic nerve activity is reduced in subjects diagnosed with Anderson-Fabry Disease (AFD) [31]. These preliminary findings were further corroborated on a larger scale in 25 patients with genetically proven AFD: in a comparison of ^123^I-mIBG and cardiac magnetic resonance imaging, impaired sympathetic nerve function preceded myocardial structural damage, such as fibrosis [32]. Apart from that, ^123^I-mIBG is also considered as a valuable risk stratification tool in non-cardiac diseases, such as neurogenerative disorders: it has been proven that HMR based on ^123^I-mIBG scintigraphy can differentiate between Dementia with Lewy Bodies and Alzheimer disease even in patients without parkinsonism [33].

## PET imaging agents for myocardial SNS assessment—^11^C-labeled tracers

Despite its potential incremental value over conventional clinical parameters, ^123^I-mIBG suffers from several drawbacks. HMR is a global marker of cardiac nerve integrity, and thus it does not provide any further details of regional heterogeneity of denervated myocardium in viable border zones [10, 34]. However, cardiac neuronal PET imaging agents offer several advantages over ^123^I-mIBG SPECT including improved spatio-temporal resolution and quantification approaches [35]. Hence, in contradistinction to a global assessment of the cardiac nerve integrity using ^123^I-mIBG-derived HMR, PET-based assessment of denervated myocardium opens avenues for regional analysis of different myocardial areas simultaneously (e.g., infarct zone, border zone or remote myocardium) [10].

Several ^11^C-labeled catecholamine analogue radiotracers have been investigated to assess myocardial SNS: The three most commonly used radiotracers are ^11^C-hydroxyephedrine (^11^C-HED), ^11^C-epinephrine and ^11^C-phenylepinephrine [12, 35]. All of these carbon-11 compounds differ in their kinetic properties, such as their affinity for neuronal uptake-1 as well as susceptibility to degrading enzymes, e.g. monoamine oxidase (MAO) and catechol-*O*-methyltransferase [36]. Hence, a combination of multiple PET radiotracers with such distinct differences in their properties may offer more profound insight into cardiac sympathetic neurotransmission in the failing heart [10, 35]: in an ^11^C-labeled triple-radiotracer approach in otherwise healthy heart-transplant recipients, different underlying characteristics in catecholamine handling after cardiac transplantation was demonstrated [37].

In clinical trials and daily clinical routine, ^11^C-HED is the most widely used PET radiotracer to assess sympathetic nerve conditions: in a recent study, a low global ^11^C-HED retention was linked to poorer overall survival in patients with LV dysfunction [38]. Analogous to its scintigraphy-based counterpart (ADMIRE-HF) [17], the prospective “Prediction of ARrhythmic Events with Positron Emission Tomography (PAREPET)” trial proved that the volume of denervated myocardium is a strong predictor of sudden cardiac arrest, independent of other clinical parameters, such as LVEF, infarct volume or hybernating myocardium. Quantitatively, each 1% increase in the denervated myocardial volume was associated with a 5.7% increase in sudden cardiac arrest. Hence, this PET-driven imaging assessment of myocardial sympathetic denervation could also be helpful in differentiating between low- and high-risk patients who most likely benefit from ICD implantation [39]. As a common comorbidity in chronic heart disease [40], Hall et al. investigated the beneficial use of ^11^C-HED in HF patients suffering from obstructive sleep apnea (OSA) under continuous positive airway pressure (cPAP) therapy. Of note, short-term cPAP therapy of 6–8 weeks increased ^11^C-HED retention and, therefore, indicated an improved myocardial SNS. This might pave the way to link potential alterations in ^11^C-HED retention indices to clinical outcome in patients suffering from OSA [41].

## Novel cardiac neuronal PET imaging agents: ^18^F-labeled radiotracers

^11^C-labeled radiotracers are currently the most frequently used cardiac PET imaging agents for mapping sympathetic nerve conditions of the heart. However, higher expenses for purchase, maintenance of costly on-site cyclotrons, and the necessity of staff to prepare such radiopharmaceuticals increases the financial burden for both patients as well as for primary or secondary care hospitals. The financial burden for radiotracer production is a consideration for practitioners as to what extent ^11^C-HED should be employed in daily clinical routine [26]. On the other hand, ^18^F-labeled radiotracers have a significantly longer half-life (110 min) and might overcome these hurdles. First, the longer half-life allows for delivery from central cyclotron facilities to smaller hospitals with stand-alone PETs, which has proven to be cost-effective for the commonly used oncology imaging agent 2-deoxy-2-^18^F-fluoro-d-glucose [42]. Such ^18^F-labeled cardiac neuronal imaging agents could even be distributed by commercial vendors [43]. Second, the longer half-life would also allow for flexibility in the study protocol, e.g. by facilitating the acquisition of both early and delayed time point imaging analogous to ^123^I-mIBG. Moreover, fluoride introduction into PET agents increases the stability of a radiopharmaceutical, as the risk of metabolism at sensitive positions is reduced [44]. Consequently, ^18^F-labeled radiotracers can help realize the full potential of PET imaging technology and, therefore, novel cardiac SNS imaging agents have recently been introduced, including ^18^F-LMI1195 as well as ^18^F-Fluoro-Hydroxyphenethylguanidines [^18^F-fluoro-3-hydroxyphenethylguanidine (^18^F-4F-MPHG) and its structural isomer 3-^18^F-fluoro-4-hydroxyphenethylguanidine (^18^F-3F-PHPG)] [44].

### Fluorobenzylguanidine (^18^F-LMI1195)

Sharing a benzylguanidine structure similar to its SPECT counterpart ^123^I-mIBG, ^18^F-LMI1195 is not subject to further metabolism by enzymes such as MAO. As a major advantage, it can be easily obtained by a simple one-step ^18^F replacement reaction [44].

Using a cell membrane-binding assay, it was demonstrated that ^18^F-LMI1195 is taken up via uptake-1 mechanism into the presynaptic nerve terminal. Moreover, in an in vitro blocking study using the potent uptake-1 blocking agent desipramine (DMI), the high specificity of ^18^F-LMI1195 to uptake-1 was further corroborated [45]. Utilizing isolated perfused rabbit hearts to avoid systemic recirculation and metabolism of the radiotracer, a flow-dependent decrease of the first-pass radiotracer extraction fraction was demonstrated: therefore, one might speculate that the initial uptake depends on the radiotracer quantity in the extracellular spaces which is limited by blood flow [46]. However, Yu et al. examined a denervated rabbit model and after an initial uptake in the first 2 min in both innervated and denervated areas, ^18^F-LMI1195 was rapidly washed out thereafter [47].

**Fig. 2:**
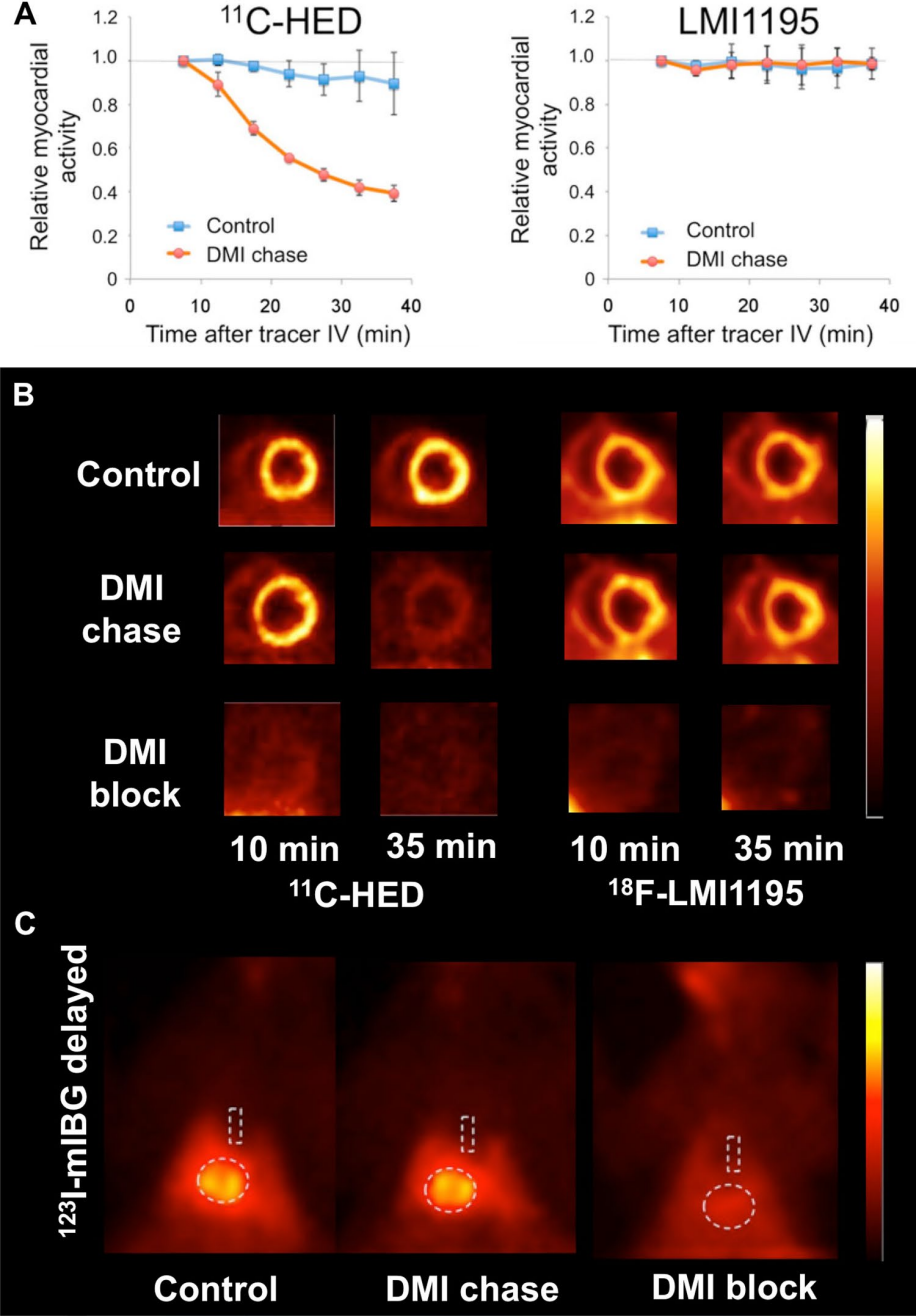
**a** Averaged time activity curves and **b** representative short-axis images of in vivo rabbit cardiac PET imaging. Desipramine (DMI) chase (i.e., DMI administered after tracer delivery) enhanced ^11^C-Hydroxyephedrine (^11^C-HED) tracer washout, while ^18^F-LMI1195 washout remained stable. **c** Results of in vivo rabbit ^123^I-metaiodobenzylguanidine (^123^I-mIBG) planar scintigraphy of the chest. Similar to ^18^F-LMI1195, DMI chase did not change cardiac distribution of ^123^I-mIBG. This might be due to the underlying benzylguanidine structure shared by both radiotracers. Dotted lines indicate regions of interest in both heart and mediastinum. Modified from Werner et al. [35] © by the Society of Nuclear Medicine and Molecular Imaging, Inc.

After being taken up into presynaptic nerve terminals via uptake-1, it was questioned whether ^18^F-LMI1195 is stored in presynaptic vesicles [10]. Therefore, in an in vivo head-to-head comparison using healthy rabbits, the most commonly used SPECT/PET catecholamine analogue radiotracers (^123^I-mIBG, ^11^C-HED) and ^18^F-LMI1195 were studied by our research group: all of these tracers could be blocked by pretreatment with the potent uptake-1 blocker DMI before tracer injection. Similar findings of a selective NET inhibitor blockage via DMI has been previously reported for both ^123^I-mIBG and ^18^F-LMI1195 in the rabbit heart [45]. However, distinct radiotracer properties were revealed using a DMI chase protocol (i.e., DMI administered after tracer delivery): retention kinetics of the benzylguanidine-based tracers ^123^I-mIBG and ^18^F-LMI1195 remained stable, while ^11^C-HED washout increased significantly. Integrating these data, it can be hypothesized that ^123^I-mIBG and ^18^F-LMI1195 are stably stored in presynaptic vesicles (resistant to NET inhibitor chase) and, therefore, mimic physiological NE turnover. On the contrary, ^11^C-HED undergoes a continuous cycle of NET uptake and release at the nerve terminal (Fig. 2) [35]. These findings were further corroborated by electric field stimulation in isolated perfused rabbit hearts: this approach lead to increased washout rate of ^18^F-LMI1195, as it provoques vesicular NE release [46]. Of note, all of those studies were conducted using rabbit myocardium: similar to the human heart, the contribution of NE clearance to neuronal uptake-1 is also pronounced in rabbits and, therefore, the rabbit heart serves as a suitable platform to mimic human cardiac nerve status [48, 49]. However, our research group also recently investigated (vesicle-poor) SK-N-SH cells vs. (vesicle-rich) PC12 cells in combination with ^18^F-LMI1195 and we found that stimulants for storage vesicle turnover (reserpine, high potassium chloride) enhanced ^18^F-LMI1195 washout from PC12 cells, while radiotracer retention remained stable in SK-N-SH cells [50].

Multiple studies have reported on the high specificity of ^18^F-LMI1195 for uptake-1 in different species, such as monkeys. A clear delineation of the myocardium was visualized with a significantly higher heart-to-background ratio, mainly due to a rapid washout from the lung and liver as well as a quick radiotracer clearance from the blood. Compared to ^123^I-mIBG, a three-fold higher heart-to-liver ratio could be derived for ^18^F-LMI1195 in cynomonkeys [45]. However, species- and radiotracer-dependent variations among different cardiac catecholamine analogue tracers have to be taken into account. For example in Wistar rats, cardiac uptake of ^18^F-LMI1195 was significantly altered using the NE uptake-2 inhibitor phenoxybenzamine (but not by the uptake-1 blocker DMI). Thus, in contradistinction to other species, ^18^F-LMI1195 is primarily a substrate of uptake-2 in rats [51]. However, ^11^C-HED can be successfully inhibited by the uptake-1 blocker DMI in rat hearts and, therefore, ^11^C-HED uptake has a high affinity to neuronal uptake-1 in that species. Of note, this does not apply to ^123^I-mIBG: analogous to ^18^F-LMI1195, ^123^I/^131^I-mIBG demonstrated distinct characteristics of uptake mechanism that are compatible with a significant contribution from nonneuronal uptake-2 in the rat heart [52]. The underlying chemical structure (benzylguanidine) might explain the similarities between mIBG and LMI1195 in the rat myocardium. Hence, to investigate catecholamine analogue radiotracer handling in the nerve terminal, the animal platform to test a particular SNS imaging agent has to be chosen with extreme caution [52]. ^18^F-LMI1195 might also allow for detection and quantification of loco-regional areas of denervation. In denervated rabbit myocardium, ^18^F-LMI1195 could differentiate between innervated vs. denervated areas, while the reference myocardial perfusion PET radiotracer ^18^F-flurpiridaz demonstrated no abnormalities. Of note, ^18^F-LMI1195 was able to monitor reinnervation in previous denervated areas (2 vs. 12 weeks post-denervation) [47].

**Fig. 3:**
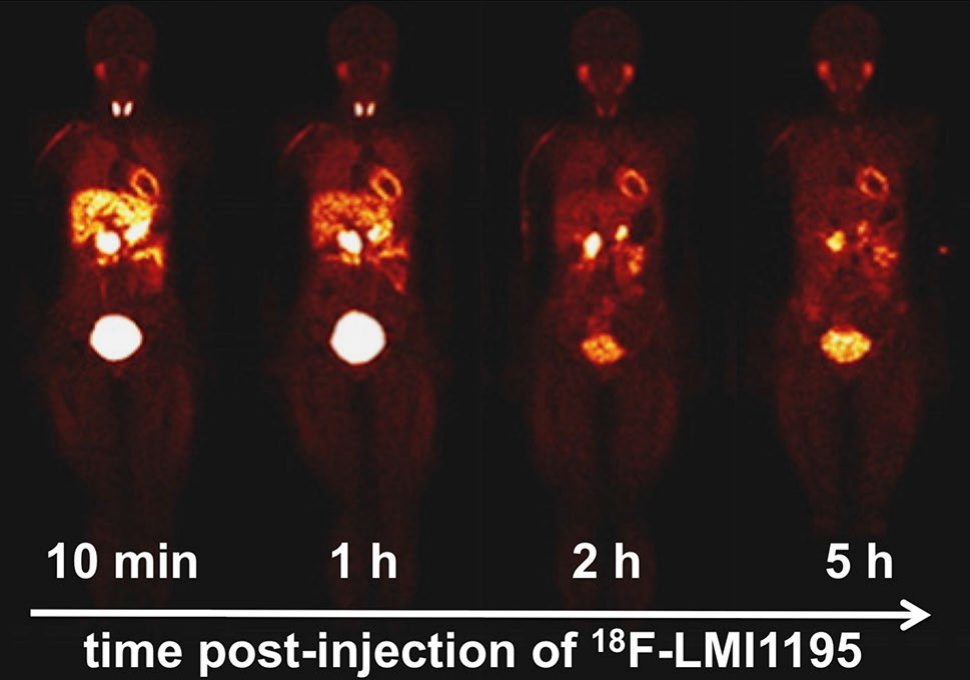
Representative sequences of whole-body ^18^F-LMI1195 images in healthy volunteers approximately 10 min, 1, 2 and 5 h post-injection. Modified from Sinusas et al. [53] © by the Society of Nuclear Medicine and Molecular Imaging, Inc.

Noteworthy, ^18^F-LMI1195 is the only ^18^F-labeled catecholamine analogue radiotracer that has entered phase-1 and phase-2 trials. Sinusas et al. reported on promising radiotracer characteristics with favorable biodistribution: over 5 h, stable myocardial activity, which was uniform throughout the myocardium, as well as rapid blood clearance was demonstrated. Dynamic PET revealed excellent heart-to-lung and heart-to-liver ratios (Fig. 3). In the twelve healthy volunteers, no adverse events or safety concerns were documented. Dosimetrically, the most critical organ was identified as the urinary bladder wall, followed by the kidneys. However, the mean effective dose was rather low and comparable to ^123^I-mIBG [53]. Of note, in a recently published head-to-head comparison of ^18^F-LMI1195 and ^11^C-HED, preliminary assessment in 9 participants revealed comparable estimates of cardiac autonomic nervous function. However, compared to its ^11^C-based counterpart, the ^18^F-labeled imaging agent demonstrated superior kinetics, in particular for early cardiac imaging [54]. Hence, this phase-2 trial might pave the way for a phase-3 trial, which would further investigate the potential benefit of ^18^F-LMI1195 for risk stratification among HF patients.

### ^18^F-fluoro-hydroxyphenethylguanidines

Apart from ^18^F-LMI1195, two other ^18^F-labeled catecholamine analogue radiotracers have been introduced, namely ^18^F-4F-MPHG and ^18^F-3F-PHPG. Both radiotracers were taken up more slowly and demonstrated significantly longer neuronal retention times compared to ^11^C-HED or ^11^C-mIBG in isolated rat hearts, mainly due to efficient storage inside NE storage vesicles [55]. Such key characteristics might be of utmost importance in kinetic analyses of regional nerve density assessment, as such radiotracers are capable of reflecting even modest alterations of cardiac sympathetic nerve conditions without being hampered by the blood flow effect. Common sympathetic nerve agents such as ^11^C-HED or ^123^I-mIBG are too rapidly taken up via uptake-1 to allow for reliable compartmental modeling of their kinetics. Thus, the neuronal uptake for ^11^C-HED by uptake-1 from extracellular space (k3) is much faster than k2 (clearance from extracellular space back to plasma), i.e., k3 ≫ k2. Consequently, ^11^C-HED retention measurements are not sensitive to moderate loss in nerve integrity and rather limited to myocardial regions in which nerve losses are severe [43]. However, the phenethylguanidine-like structure of ^18^F-4F-MPHG and ^18^F-3F-PHPG overcomes these hurdles as they offer the main advantage of slow neuronal uptake and long neuronal retention [55].

In-vivo imaging studies in monkeys further corroborated the favorable kinetic properties of both ^18^F-labeled radiotracers, as the myocardium could be clearly delineated. In an in vivo blocking study with varying amounts of DMI, retention indices of ^18^F-4F-MPHG decreased with increasing levels of the uptake-1 blocking agent. The obtained “net uptake rate” constant, derived from compartmental modeling or Patlak analysis of ^18^F-4F-MPHG kinetics, also declined dose-dependently with increasing DMI doses [56]. Thus, these compounds seem to have optimal kinetics and can allow for quantitative assessment of even a slight loss of cardiac sympathetic nerve density or regional denervation [44].

^18^F-4F-MPHG and ^18^F-3F-PHPG have been tested in humans and both imaging agents demonstrated clear delineation of the myocardium along with low liver and lung uptake. Kinetic analyses of both radiotracers using Patlak demonstrated encouraging results and regional mapping of cardiac nerve integrity in humans seems feasible [57].

Table 1 summarizes the herein presented SPECT and PET catecholamine analogue tracers to assess cardiac sympathetic nerve conditions, along with several advantages and limitations of each agent.

**Table 1:** Head-to-head comparison of single-photon emission computed tomography (SPECT) vs. positron emission tomography (PET) agents for cardiac innervation imaging, along with several advantages and limitations

Imaging Modality	Catecholamine analogue tracer	Advantages	Limitations
SPECT	^123^I-mIBG	Long-standing experience [13] Food-and-drug administration approved [11] Considered as the “work horse” [13, 26] Clincially well-established HMR ratio [15] Evidence from different prospective trials (ADMIRE-HF, ADMIRE-ICD) [17]	HMR as a global marker of cardiac nerve integrity [10] No regional analysis of different myocardial areas [10, 44] HMR primarily obtained by planar images [15] Cost-effectiveness data are lacking [13]
PET	^11^C-Hydroxyephedrine	Higher spatio-temporal resolution [11] Most widely used cardiac sympathetic nerve PET radiotracer [11] Denervated myocardium as a strong predictor of sudden cardiac arrest (PAREPET trial) [39] Quantitative assessment of different myocardial areas [44]	High financial burden [44] Short half-life of ^11^C (20.4 min) limits the design of novel imaging agents [44] Potential cold-mass effect of ^11^C [59] Phase-2 study: superior kinetics of ^18^F-LMI1195 compared to ^11^C-HED [54]
	^18^F-LMI1195	Simple one-step ^18^F replacement reaction [44] First ^18^F-labeled radiotracer in a phase-1 and -2 trial [53, 54] Longer half-life (110 min): flexibility in the study protocol, higher flexibility in producing novel SNS cardiac imaging agents [11, 44] Readily transported and stored into synaptic vesicles [35] Phase 2 study: superior kinetics of ^18^F-LMI1195 [54]	Limited to university hospitals/tertiary referral hospitals No data available regarding potential benefit in risk stratification among HF patients
	^18^F-Fluoro-Hydroxyphenethyl-guanidines (^18^F-4F-MPHG and ^18^F-3F-PHPG)	Slow uptake and longer neuronal retention times: potential to reflect even modest alterations of cardiac sympathetic nerve conditions [55] Feasibility of compartmental modeling or Patlak analysis [55, 57] First-in-human study: low liver and lung uptake [57]	Limited to university hospitals/tertiary referral hospitals No data available regarding potential benefit in risk stratification among HF patients

AdreView myocardial imaging for risk evaluation in heart failure (ADMIRE-HF), International Study to determine if AdreView heart function scan can be used to identify patients with mild or moderate heart failure (HF) that benefit from implanted medical device (ADMIRE-ICD), heart-to-mediastinum ratio (HMR), Prediction of ARrhythmic events with positron emission tomography (PAREPET)

^*123*^*I-mIBG* ^123^I-meta-Iodobenzylguanidine, ^*11*^*C-HED* ^11^C-hydroxyephedrine, ^18^F-LMI1195 N-[3-Bromo-4-(3-[^18^F ]fluoro-propoxy)-benzyl]-guanidine, ^*18*^*F-4F-MPHG* ^18^F-fluoro-3-hydroxyphenethylguanidine, ^*18*^*F-3F-PHPG* 3-^18^F-fluoro-4-hydroxyphenethylguanidine

## Conclusions

In this review, we provided an overview of the most commonly used SPECT and PET catecholamine analogue radiotracers for mapping myocardial nerve integrity. ^123^I-mIBG is considered to be the “Work Horse” in daily clinical routine [13] and multiple studies have demonstrated its benefit in different clinical scenarios. The prospective ADMIRE-HF trial showed its prognostic capability in identifying HF patients at risk, independent of other clinical parameters such as LVEF [17]. Moreover, the currently event-driven recruiting ADMIRE-ICD trial will determine the role of this imaging approach in selecting appropriate candidates for left-ventricular assistant device therapy [26]. However, ^123^I-mIBG merely provides a global assessment of cardiac nerve integrity, but novel innovative techniques such as Cadmium-zinc-telluride scanners may provide further information beyond available standard planar imaging techniques [58]. In contradistinction to SPECT, neuronal PET imaging agents offer several advantages, such as improved spatio-temporal resolution or regional analysis of different myocardial areas [10]. Multiple ^11^C-labeled catecholamine analogue PET radiotracers have been investigated to assess myocardial SNS: in particular, the prospective PAREPET trial demonstrated a strong association between the volume of denervated myocardium assessed by ^11^C-HED and cardiac events [39]. Compared to their ^11^C-labeled counterparts, the longer half-life of ^18^F allows for delivery from central cyclotron facilities to smaller hospitals with stand-alone PET scanners and, consequently, such ^18^F-labeled cardiac imaging agents could even be distributed by commercial vendors. Thus, a costly on-site cyclotron is not needed and the financial burden for the healthcare system may be significantly reduced. Moreover, ^18^F-labeled radiotracers also allow for more flexibility in the study protocol, such as delayed imaging [44]. The ^18^F-labeled imaging agents included in this review (^18^F-LMI1195, ^18^F-4F-MPHG and ^18^F-3F-PHPG) demonstrate excellent imaging quality and key properties such as reduced background uptake due to favorable heart-to-liver ratios, regional assessment of different myocardial areas, and/or kinetic behavior at the nerve terminal similar to physiological NE [35, 57]. Encouraging results from a recently published phase-2 trial demonstrated that the kinetics of ^18^F-LMI1195 might be superior to ^11^C-HED: this could pave the way for a phase-3 trial to evaluate the potential role of ^18^F-LMI1195 for risk stratification among HF patients [54]. On the contrary, ^18^F-4F-MPHG and ^18^F-3F-PHPG offer the advantage of slow neuronal uptake and long neuronal retention and, therefore, such radiotracers are capable of reflecting even modest alterations of cardiac sympathetic nerve conditions by kinetic analyses [55]. Nonetheless, future studies should focus on the underlying mechanisms of these PET and SPECT agents, in particular to guide the clinical reader in interpreting imaging results or to combine multiple radiotracers with different kinetic properties to gain further insight into NE handling at the nerve terminal.
